# Intracerebral Adenosine During Sleep Deprivation: A Meta-Analysis and New Experimental Data

**DOI:** 10.5334/jcr.171

**Published:** 2018-10-09

**Authors:** Cathalijn H. C. Leenaars, Sergey A. Savelyev, Stevie Van der Mierden, Ruud N. J. M. A. Joosten, Maurice Dematteis, Tarja Porkka-Heiskanen, Matthijs G. P. Feenstra

**Affiliations:** 1 RadboudUMC, Utrecht University, NL; 2 Hannover Medical School, DE; 3 Medical Biological Research and Development “Cytomed”, RU; 4 The Netherlands Brain Institute, NL; 5 Grenobles Alpes University Hospital and Grenoble Alpes University, FR; 6 University of Helsinki, FI

**Keywords:** sleep deprivation, adenosine, medial prefrontal cortex, AMP, meta-analysis

## Abstract

The neuroregulator adenosine is involved in sleep-wake control. Basal forebrain (BF) adenosine levels increase during sleep deprivation. Only a few studies have addressed the effect of sleep deprivation on extracellular adenosine concentrations in other brain regions.

In this paper, we describe a microdialysis experiment as well as a meta-analysis of published data. The 64 h microdialysis experiment determined the extracellular adenosine and adenosine monophosphate (AMP) concentrations in the medial prefrontal cortex of rats before, during and after 12 h of sleep deprivation by forced locomotion. The meta-analysis comprised published sleep deprivation animal experiments measuring adenosine by means of microdialysis.

In the animal experiment, the overall median adenosine concentration was 0.36 nM and ranged from 0.004 nM to 27 nM. No significant differences were observed between the five conditions: 12 h of wash-out, baseline light phase, baseline dark phase, 12 h of sleep deprivation and 12 h of subsequent recovery. The overall median AMP concentration was 0.10 nM and ranged from 0.001 nM to 7.56 nM. Median AMP concentration increased during sleep deprivation (T = 47; p = 0.047) but normalised during subsequent recovery.

The meta-analysis indicates that BF dialysate adenosine concentrations increase with 74.7% (95% CI: 54.1–95.3%) over baseline during sleep deprivation. Cortex dialysate adenosine concentrations during sleep deprivation were so far only reported by 2 publications.

The increase in adenosine during sleep deprivation might be specific to the BF. At this stage, the evidence for adenosine levels in other brain regions is based on single experiments and insufficient for generalised conclusions. Further experiments are currently still warranted.

## Introduction

Adenosine is a purine nucleoside involved in the cellular energy metabolism [1,2]. In addition, it is a neuroregulator involved in sleep-wake control [2,3]. Caffeine is the most commonly ingested nonselective adenosine antagonist; an estimated 85% of US population, and nearly one third of the adolescents, drink one or more caffeinated beverages each day [4]. When sleep is restricted, caffeine is an effective strategy to counteract sleepiness and maintain physical and cognitive performance [5]. With normal doses and in healthy adults, it has no health risks. However, while caffeine hardly affects sleep quality in infants, it can impair sleep quality and result in daytime sleepiness in the morning in adolescents and adults [4].

Brain mechanisms underlying wake and sleep seem to be strongly conserved throughout evolution [6], indicating their importance, but also underscoring the value of animal studies in clarifying the neuroanatomical organisation of the sleep-wake cycle. This is convenient, as measurements of adenosine in the human brain are rare; we are aware of only one human microdialysis study [7].

The basal forebrain (BF) may contain the core mechanism for sleep-wake control [8]. BF GABAergic neurons promote sleep [9], and sleep homeostasis is maintained by changes in extracellular BF adenosine[10]. The need for sleep increases during the waking period, parallel to accumulation of adenosine in the BF. Upon reaching a certain threshold, adenosine promotes the transition from wake to nonrapid eye movement sleep [11]. Next to the sleep-promoting BF, the ascending reticular activating system, projecting to the cortex directly or via the thalamus, may be the main system for maintenance of wakefulness [12].

While the effects of adenosine on increasing sleep pressure seem specific to the basal forebrain, the release of adenosine may be more generalised throughout the brain. As a side-product of energy metabolism, it could be released non-specifically with all neuronal activity. Several animal experiments have shown increases in BF adenosine during sleep deprivation [13,14,15,16,17]. Only a few studies have addressed the effect of sleep deprivation on extracellular adenosine concentrations in other brain regions. We hypothesise that the observed increases in adenosine are specific to the basal forebrain.

The prefrontal cortex (PFC) may be particularly sensitive to sleep [18]. In the human brain, prominent deactivation of prefrontal regions occurs during sleep [19] and during sleep deprivation [20]. The medial prefrontal cortex (mPFC) region is involved in generation of slow wave sleep [21]. Besides, sleep seems specifically necessary for PFC-dependent tasks. For example, attention, working memory, temporal memory and behavioural inhibition are affected by sleep deprivation [18]. PFC-dependent task performance is also affected by sleep deprivation in animals, e.g. on tasks addressing working memory and task switching [22,23].

Extracellular adenosine accumulates in the BF with prolonged wakefulness; it also could accumulate in the cortex. In humans, mPFC adenosine A1 receptors are upregulated during sleep deprivation [24]. However, not all types of mPFC neurons are affected by adenosine [25]. Thus, it would be specifically interesting to know what happens to extracellular adenosine within the prefrontal cortex.

Microdialysis is a convenient method to study adenosine in vivo, based on simple diffusion; adenosine diffuses from the extracellular space through a semi-permeable membrane into a continuously flowing isotonic fluid. Preceding studies measured adenosine in the lateral gyrus [26], frontal associative cortex [27] and cingulate cortex [27]. In this paper we describe an experiment determining the effect of 12 h of sleep deprivation on mPFC adenosine concentrations. Besides, we performed a meta-analysis of sleep deprivation studies [28].

Meta-Analyses are common practice in clinical research, but so far less common for animal studies. A meta-analysis performed in combination with a full systematic review (SR) is considered to provide the highest possible level of evidence to answer a research question. We recently completed an SR analysing adenosine and AMP baseline concentrations in microdialysates in relation to the experimental design [28]. In that review, we included several studies on the effect of sleep deprivation on adenosine, which are analysed in this paper.

## Materials and Methods

### mPFC microdialysis experiment

The microdialysis experiment in rats, before, during and after sleep deprivation by means of forced locomotion, has been described previously [29]. In short, 11 Wistar rats were prepared for microdialysis with custom-made concentric microdialysis probes (e.g. [30], exposed membrane length: 4 mm). Probes were placed into the mPFC at an angle of 12° (AP + 3.0 mm; L ± 1.8 mm; V–5.5 mm; relative to Bregma).

After approximately 1 week of post-surgical recovery, the experiment started. Rats were connected to the microdialysis set-up and placed into separate compartments of 2 adjacent sleep deprivation devices. Artificial cerebrospinal fluid (145 mmol/l NaCl, 1.2 mmol/l CaCl_2_, 2.7 mmol/l KCl, 1.0 mmol/l MgCl_2_) was perfused through the probes at a flow rate of 3 μl/min.

After 12 h of habituation to the experimental environment, sampling continued for 24 h of baseline measurements. This was followed by 12 h of sleep deprivation during the light phase (modelling a sleepless night in humans) and subsequent recovery for 16 h. Food and water were available *ad libitum* throughout the experiment. The experiment was approved by the experimental animal committee of the Royal Netherlands Academy of Arts and Sciences and performed in accordance with national standards of care.

### Microdialysis sampling

Dialysate was collected in 1 h samples (i.e. 180 μl) in plastic vials (300 μl; 7431100, Aurora Borealis) in a refrigerated fraction collector (6°C; CMA 470, Aurora Borealis). Samples were kept on 6°C in the refrigerated fraction collector until they were transferred to ice. Samples were split into 8 fractions and kept on ice for the remainder of the experiment. After the experiments were finished, all fractions were stored at –80°C. Corticosterone content was determined in the first 60 μl fraction [29]. The 15 μl fractions destined for adenosine measurements were transported on dry ice from Amsterdam to Helsinki. There they were again stored at –80°C until analysis. Our preceding paper only described the corticosterone concentrations, as part of the validation of our novel method for sleep deprivation [29]. Other neurochemical measurements have not yet been presented. We now describe dialysate adenosine levels. Publications of amino acids and monoamines measured in the same dialysates are in progress.

### Adenosine and AMP measurements

Adenosine and AMP were simultaneously measured using high-performance liquid chromatography (HPLC) with fluorescence detection. To render them fluorescent, adenosine and AMP underwent derivatisation with chloracetaldehyde, as described by e.g. Matuszewski and Bayne [31]. The derivatisation reagent consisted of 5 parts of 10% chloracetaldehyde, 5 parts of 1 M HCl and 1 part of 1 M ethylenediaminetetraacetic acid (EDTA). 5.5 μl of modification reagent and 60 μl of water were added to each 15 μl sample, followed by incubation at 60°C for 120 min.

The HPLC system consisted of an LC-10AD pump (Shimadzu Corporation, Kyoto, Japan), a SIL-20AC Prominence autosampler (Shimadzu) and a fluorometric detector (Waters Corporation, Milford, MA, Japan; excitation/emission wavelengths set to 265/399 nm). Chromatography was performed on a reversed phase column (Luna® 5 μm C18 (2) 100 Å, 150 × 4.00 mm; Phenomenex, USA). Compounds were isocratically eluted with mobile phase at 1 ml/min. The mobile phase contained 50 mM ammonium acetate, 0.2 mM tetrabutylammonium hydrogen sulphate, 1 M EDTA, and 15% methanol. The pH was set to 5.4–5.6 with HCl 1 M. The mobile phase was filtered (0.2 μm filter, Cat # GSTF04700, Millipore) and degassed by sonication prior to use. Concentrations were calculated from peak surface, relative to standard, with ClassVP 6.12 (Shimadzu).

### Primary data-analysis

**Figure 1:**
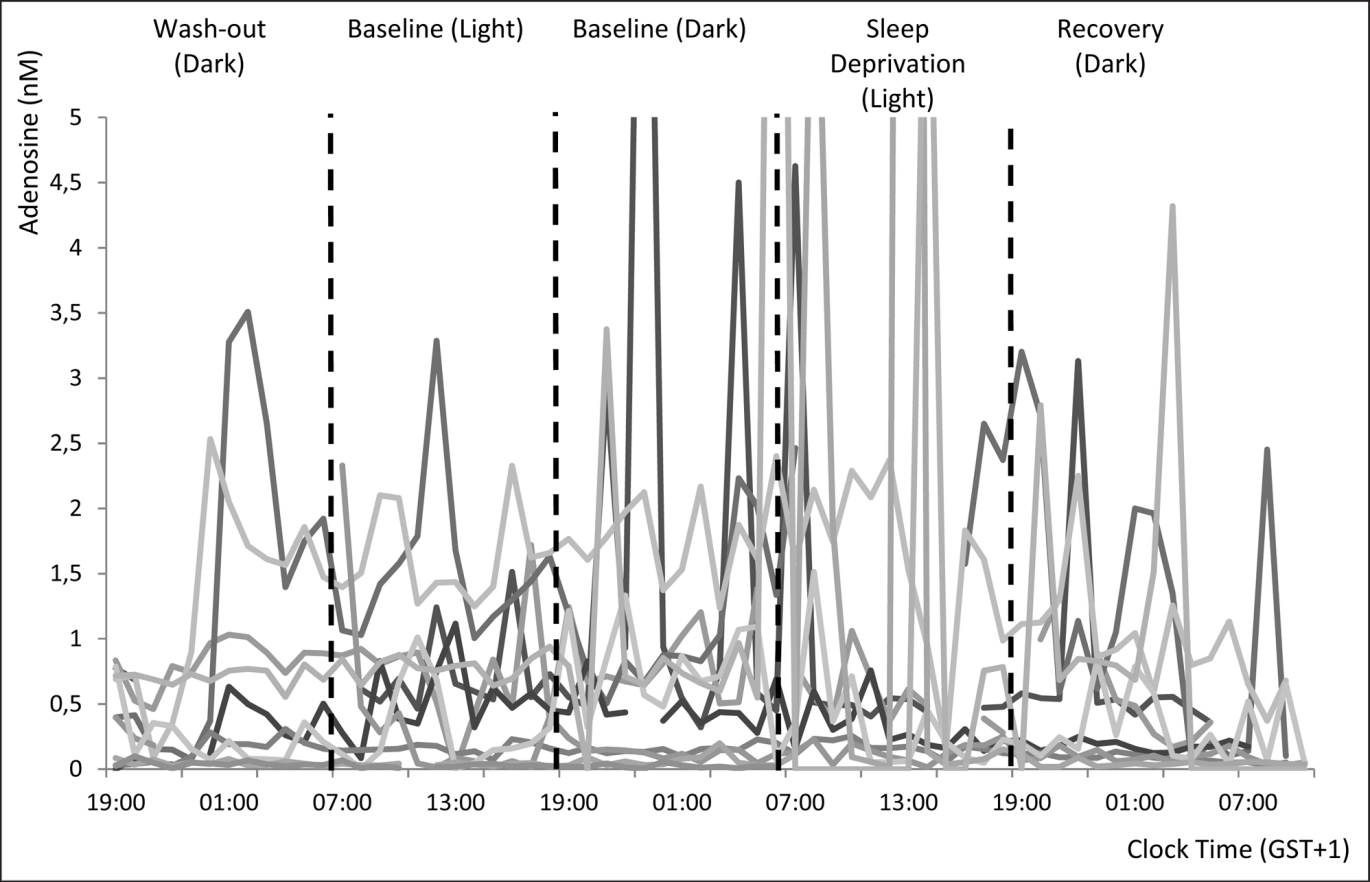
Individual adenosine concentration time curves in mPFC dialysate. N = 11. The axis was stretched to 5 nM to decrease overlap and improve visibility of individual curves, this results in 5 high values (10.0; 12.9; 7.3; 27.0; and 7.5 nM) being off the chart.

For the experiment, analyses were performed in Microsoft Excel and SPSS 22.0.0.1. Because of higher than anticipated variation (Figure 1), we selected analyses that prevent outliers affecting the results. We thus report median values and the interquartile range (IQR) instead of the more commonly used mean ± standard error. The median of each 12 h block was taken for each animal to reflect adenosine concentrations during wash-out (dark phase), baseline, sleep deprivation and the first 12 h of recovery (dark phase). Values were compared with a non-parametric Friedman repeated-measures test. When the Friedman test indicated an overall significant difference, post-hoc Wilcoxon signed rank tests were used to compare the sleep deprivation and recovery periods with their corresponding baseline.

### Meta-analysis

We previously performed an SR on adenosine concentrations in microdialysates analysing the effects of variations in the experimental design on baseline concentrations [28]. The search and inclusion criteria for the preceding review were designed to capture all studies measuring intracerebral adenosine by microdialysis in animals. To put our experimental adenosine data into perspective, while performing the full-text screening for the previous review, we identified papers in which microdialysate adenosine was measured during sleep deprivation. For the current review and meta-analysis, we included papers using experimental sleep deprivation or sleep interruption only. We excluded papers addressing adenosine levels during naturally occurring sleep.

Literature data were available for the BF, brainstem, cortical regions, hypothalamus, nucleus accumbens and thalamus. Adenosine concentrations in the brainstem [26], hypothalamus [26], nucleus accumbens [15] and thalamus [26] were only reported in one paper and therefore not meta-analysed. Data-extraction followed our preceding review. Additionally, we extracted the following variables: duration and type of sleep deprivation, and percentage of baseline adenosine concentration during sleep deprivation. Universal Desktop Ruler (AVPSoft) was used when adenosine data were only available graphically.

As several of the included papers did not report the absolute adenosine concentrations, all data were analysed as percentage of baseline and the corresponding standard errors of the mean. Where necessary, average values and corresponding standard deviations were converted. We performed random-effects meta-analyses on the averaged within-subject change scores. If data were provided for multiple time points during sleep deprivation, we selected the last time point (with the expected maximum effect) for inclusion in the analyses.

Our first meta-analysis assessed the effect of sleep deprivation on BF microdialysate adenosine. One reference provided data from separate groups of sleep fragmented animals [15]. These groups were included in the analyses as if they were separate studies. BF data were thus available for 10 experimental groups. We used the metagen function from the meta package to compare within-subject changes, in R 3.2.4 [32] via RStudio, as described before [33].

Our second meta-analysis assessed the difference in changes in microdialysate adenosine between cortical regions and the BF. Data from cortical adenosine were available from two references [26,27]. We used the metacont function from the meta package to compare continuous outcome data for the experimental groups within studies. We did not include our own experimental results in this meta-analysis, as we did not have data from the basal forebrain.

Publication bias was not formally analysed, as the number of included studies was deemed too low to provide informative results.

### Risk of Bias analysis

For the studies included in the meta-analysis, a risk of bias assessment was performed by one of the authors (CL). We used the tool developed by SYRCLE to assess Risk of bias in animal studies [34]. The tool was modified by excluding the items “allocation concealment” (selection bias, item 3), “random housing” (performance bias, item 4) and “blinding” (performance bias, item 5). As reported by Pires et al. before, these items cannot readily be implemented in sleep deprivation studies [35]. The resulting risk of bias is inherent when studying the effects of sleep deprivation. Furthermore, we excluded “selective outcome reporting” (attrition bias, item 8). Because protocols of animal studies are hardly ever publicly available before the start of the experiment, selective reporting cannot be reliably assessed.

Even though we focussed on within-subject comparisons, we kept the other items, addressing selection bias (sequence generation, item 1 and baseline characteristics, item 2). The included studies usually comprised multiple groups, and these items are informative on the sample included.

For other sources of bias (item 10), we assessed specific elements of the microdialysis technique. These comprise histological confirmation of probe location, sample storage and time to analysis, correction for the dead volume, side of the brain, etc. For incomplete outcome data (item 8) we would only have scored a low risk of bias if the authors reported on exclusion of both animals and individual samples.

We added a new item to reflect the risk of bias caused by meta-analysts extracting data from figures using digital rulers. This cannot be performed in a blinded manner. While data extraction with digital rulers may previously have been scored as lack of “Blinding” (detection bias, item 7), we chose to make a distinction, as the origin of the potential bias is different.

## Results

### Microdialysis Experiment

We aimed to collect 704 samples (64 samples of 1 h in 11 rats). The fraction for adenosine was missing for 61 fractions (8.7%) due to low or no flow for brief time periods. For one rat, the blockage was irreversible, and only wash-out and baseline data were available. Adenosine and AMP were analysed in the remaining 643 samples.

### mPFC adenosine and sleep deprivation

Adenosine was not detected in 42 out of the 643 samples (6.5%). The median adenosine concentration over the remaining 601 samples was 0.36 nM and the values found ranged from a minimum of 0.004 nM to a maximum of 27 nM. This is within the range of reported concentrations in our preceding systematic review [28]. Preliminary analyses of time curves, depicted in Figure 1, indicated high variability in some rats. The difference between minimum and maximum concentrations observed over the 64 h within individual rats ranged from 0.095 to 26.99 nM.

**Figure 2:**
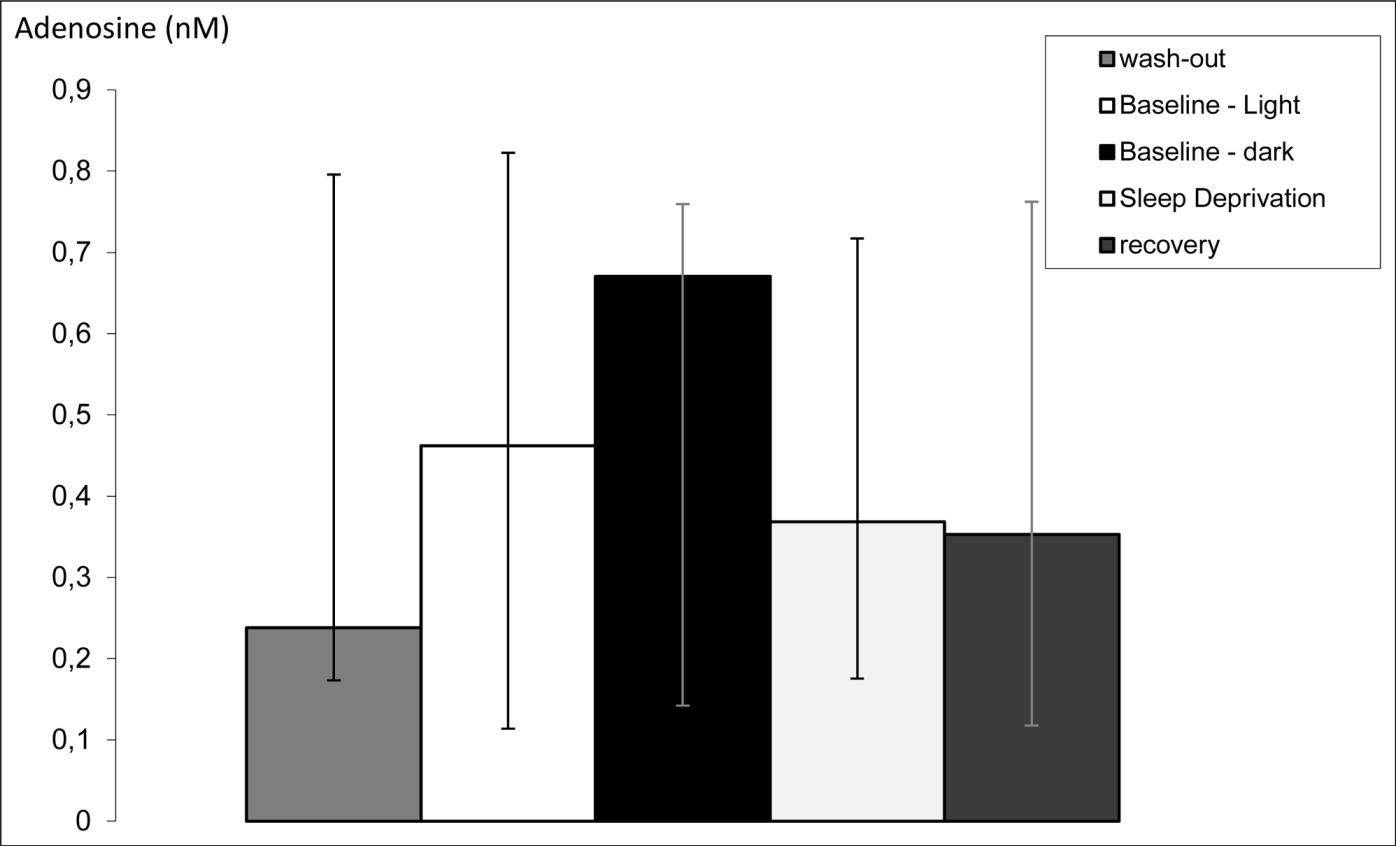
Median adenosine concentrations in mPFC dialysate with their interquartile range (IQR; n = 10–11). Concentrations are provided for the 12 h of wash-out, (IQR 0.17–0.80 nM, n = 11), baseline light phase (0.11–0.82 nM, n = 11), baseline dark phase (0.14–0.76 nM, n = 11), 12 h of sleep deprivation (0.18–0.72 nM, n = 10) and 12 h of subsequent recovery (0.12–0.76 nM, n = 10). The Friedman test indicated no significant difference between the five conditions within subjects; Fr(4) = 4.08; p = 0.395.

Median adenosine concentrations and interquartile ranges are provided in Figure 2 for the 12 h of wash-out (median 0.24 nM), baseline light phase (0.46 nM), baseline dark phase (0.67 nM), 12 h of sleep deprivation (0.37 nM) and 12 h of subsequent recovery (0.35 nM). The overall Friedman test indicated no significant difference between the five conditions within subjects; F_(4)_ = 4.08; p = 0.395. Data from the last 4 h of the experiment were not included in this analysis.

### mPFC AMP and sleep deprivation

AMP was not detected in 80 out of the 643 samples (12.4%). The median AMP concentration over the remaining 563 samples was 0.10 nM and ranged from 0.001 nM to 7.56 nM. The difference between minimum and maximum concentrations observed over the 64 h within individual rats ranged from 0.08 to 7.5 nM.

**Figure 3:**
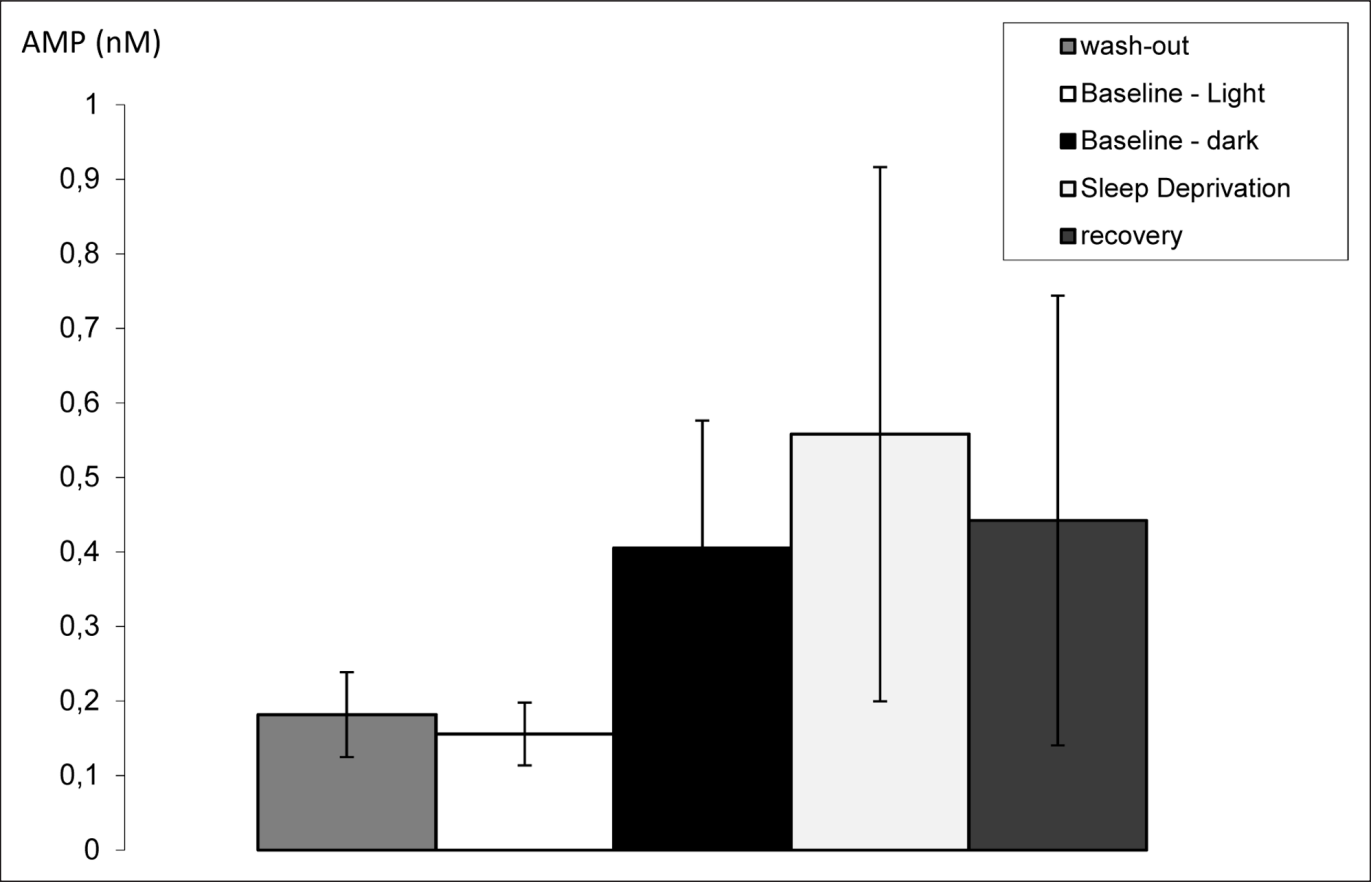
Median AMP concentrations in mPFC dialysate with their IQR (n = 10–11). AMP concentrations are provided for the 12 h of wash-out (IQR 0.01–0.15 nM, n = 11), baseline light phase (0.05–0.14 nM, n = 11), baseline dark phase (0.05–0.48 nM, n = 11), 12 h of sleep deprivation (0.06–0.64 nM, n = 10) and 12 h of subsequent recovery (0.07–0.20 nM, n = 10). Statistical details are provided in the text.

Median AMP concentrations for the 12 h of wash-out (0.05 nM), baseline light phase (0.10 nM), baseline dark phase (0.15 nM), 12 h of sleep deprivation (0.18 nM) and 12 h of subsequent recovery (0.10 nM) and the interquartile ranges are provided in Figure 3. The Friedman test indicated an overall significant difference between the five conditions within subjects; F_(4)_ = 11.4; p = 0.022. Post-hoc Wilcoxon’s signed rank tests indicated that compared to baseline, mPFC AMP increased significantly during sleep deprivation (T = 47; p = 0.047) but returned to normal during subsequent recovery (T = 21; p = 0.51). Data from the last 4 h of the experiment were not included in this analysis.

### Systematic literature review

Within our previously described sample of 132 adenosine microdialysis studies [28], we identified nine papers measuring microdialysate adenosine levels during sleep deprivation. Eight of these papers (described in Table 1) reported dialysate adenosine concentrations during sleep deprivation and prior baseline, one [36] directly compared concentrations between groups only and could not be included in the meta-analyses. Six papers reported concentrations from the basal forebrain only, two reported concentrations from the basal forebrain and the cortex.

**Table 1:** Sleep deprivation related study characteristics of studies included in the meta-analyses.

Study ID	Type of sleep deprivation	Duration of sleep deprivation (h)	Species	Brain region

Basheer, 1999[13]	Complete, Gentle Handling	3	Rat	BF
Blanco-Centurion, 2006[14]	Complete, Gentle Handling	6	Rat	BF
Kalinchuk, 2011[27]	Complete, Gentle Handling	11	Rat	BF & cortex
McKenna, 2007[15]	Sleep Interruption, forced locomotion	6 or 24	Rat	BF
Murillo-Rodriguez, 2008[16]	Complete, Gentle Handling	6	Rat	BF
Porkka-Heiskanen, 2000[26]	Complete, Gentle Handling	6	Cat	BF & cortex
Vazquez-DeRose, 2014[37]	Complete, Gentle Handling	6	Rat	BF
Wigren, 2007[17]	Complete, Gentle Handling	8.5	Rat	BF

Please note that the gentle handling method of sleep deprivation comprises several experimenter-driven strategies to keep animals awake (e.g. tapping the cages, introducing novel objects, stroking with a brush and sometimes actual handling). Only brain regions included in the meta-analyses are mentioned; either basal forebrain (BF) or BF and cortex.

### Risk of Bias in studies included in the basal forebrain and cortex meta-analyses

Reporting of the included studies was not clear enough to reliably determine risks of bias, as detailed in Table 2.

**Table 2:** Summary of risk of bias assessments.

Risk of bias item	n (of 8) included studies with		

	High risk	Low risk	Unclear risk

Sequence generation	–	–	8
Baseline Characteristics	–	–	8
Random outcome assessment	–	–	8
Blinding of outcome assessment	–	–	8
Incomplete outcome data	–	–	8
Other	–	–	8
Meta-analyst’s extraction bias	6	2	–

**Figure 4:**
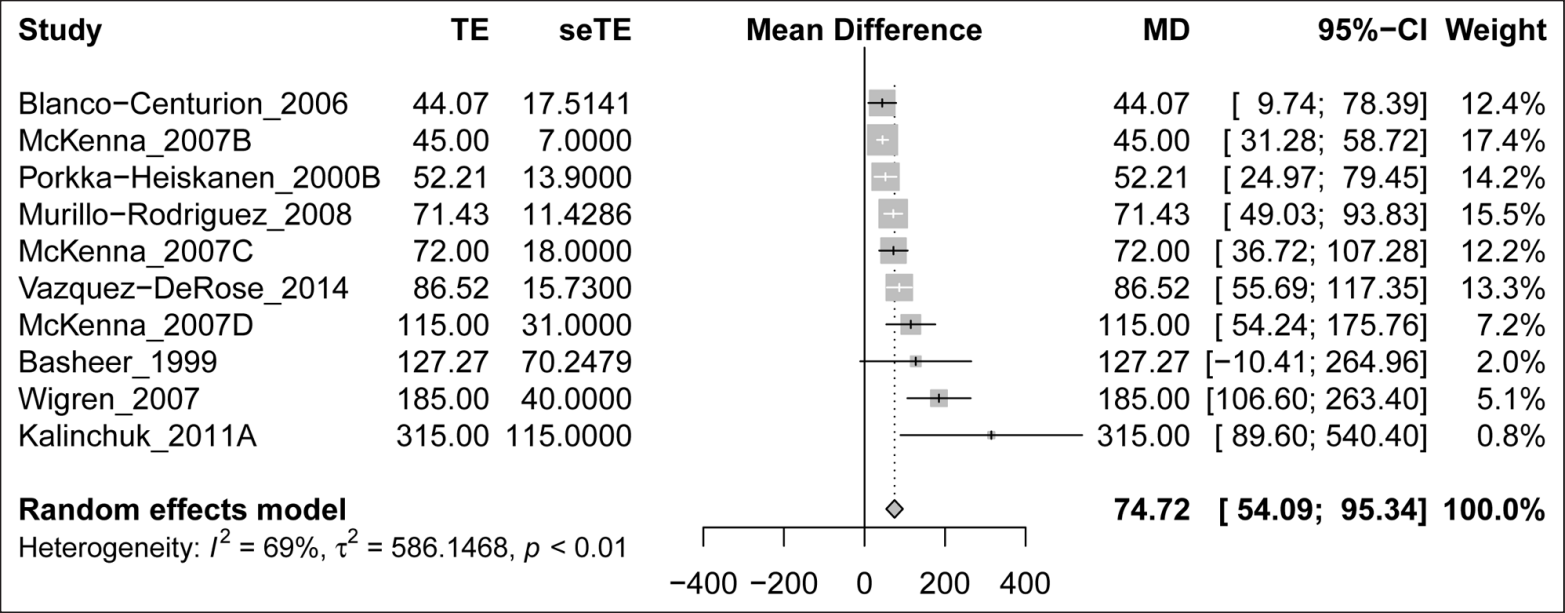
Forest plot and meta-analysis of changes in basal forebrain microdialysate adenosine concentrations during sleep deprivation. If papers provided multiple time points during sleep deprivation, we selected the last for the analyses. McKenna B, C and D refer to experimental groups exposed to different sleep deprivation protocols within the same paper (group A comprised measurements in the nucleus accumbens, not included in our meta-analyses). MD = Mean Difference (% over baseline); CI = Confidence Interval.

Our first meta-analysis indicates that in the basal forebrain, dialysate adenosine concentrations increase with 74.7% (95% CI: 54.1–95.3%) over baseline during sleep deprivation. Heterogeneity between studies is high (overall I^2^ = 68.7%). A forest plot of the included values is provided in Figure 4. We did not perform a sensitivity analysis excluding the study in cats [26] and/or the study using sleep fragmentation [15] as their values were within the range of the others.

The excluded study by Sharma et al. [36] compared the effect of sleep deprivation on basal forebrain adenosine levels between ethanol-dependent and control rats. Focussing on the control group only (n = 7), they found an increase of 31.2 ± 9.0 nM (±SEM) for the last 2 h of sleep deprivation compared to the first 2 h. No comparison with baseline was made.

**Figure 5:**
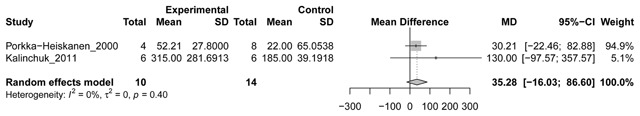
Forest plot and meta-analysis of differences between basal forebrain and cortex in changes from baseline microdialysate adenosine concentrations during sleep deprivation. If papers provided multiple time points during sleep deprivation, we selected the last for the analyses. MD = Mean Difference (% over baseline); CI = Confidence Interval.

Our second meta-analysis, provided in Figure 5, shows that averaged over only 2 studies, sleep-deprivation induced changes in adenosine levels are not significantly different between the basal forebrain and the cortex (35.28%; 95% CI: –16.03–86.60, p = 0.18)

Kalinchuck et al. [27] reported no significant changes in adenosine levels in the cingulate cortex with sleep deprivation, but as no data were shown, this study could not be included in our analysis.

## Discussion

We here present data showing an increase in AMP concentrations in the mPFC during 12 h of sleep deprivation, but not in mPFC adenosine concentrations. Based on this single experiment, we cannot know if the change in AMP is specific to the mPFC or if it also occurs in other brain regions. We imagine that the mPFC can stay active and maintain normal adenosine levels with increasing sleep pressure when resisting increasing sleep pressure. Inhibition of the synthesis of adenosine from AMP, or of AMP uptake by neurons or glial cells, could explain the observed increase in AMP during sleep deprivation. We did not observe differences in mPFC AMP or adenosine between the light and the dark phase, as was previously observed for other compounds, e.g. catecholamines [38].

Next to our experimental data, we present the first meta-analysis of the effect of sleep deprivation on extracellular adenosine levels. Our results do not contradict a preceding narrative review which concluded that the increase in adenosine during sleep deprivation is specific to the basal forebrain [39]. Overall, sleep deprivation increases adenosine in the BF, and it may or may not affect adenosine in other brain regions. However, as the difference in adenosine concentrations between BF and cortex during sleep deprivation was not significant, the overall body of evidence is not yet convincing. Of note, in the previously mentioned human study [7], adenosine was measured in the amygdala, supplementary motor area or hippocampus of 7 male epileptic patients undergoing surgery for refractory seizures. Patients were awake for 40 h, but no increases in adenosine were observed. With a maximum of n = 5 patients for a brain region (the amygdala), this single study cannot be considered conclusive.

In the BF, sleep deprivation leads to more pronounced elevations of extracellular adenosine levels than voluntary wake periods [10,40] Sleep deprivation also increases mRNA adenosine A1 receptors in the rat BF [41]. This is in line with fMRI data showing decreased cerebral metabolism in certain brain regions during sleep deprivation: prefrontal and temporal cortices, thalamus, cerebellum and basal ganglia [42]. Adenosine induces sleep either by direct inhibition of wake-promoting structures [43] or by disinhibition of GABAergic synaptic inputs of the ventrolateral preoptic nucleus [44].

Our microdialysis experiment is relatively long; while many microdialysis experiments are performed on a single working day, we sampled from individual rats for 64 h. This allowed for 12 h of wash-out and establishment of a full circadian baseline before the onset of sleep deprivation. Microdialysate adenosine concentrations had previously been reported to be stable over 4 days after probe insertion [26], and overnight wash-out protocols (usually without sampling) are not uncommon in microdialysis studies. Also, our sample size (n = 11) is high compared to previous work; group size in the studies included in our meta-analysis range from 6 to 10 animals. The success of our experiment is reflected in the previously published circadian curve of corticosterone [29].

Our experimental data were highly variable, which could have masked small changes in adenosine concentration with sleep deprivation. We cannot fully explain this high variability. We limited the effect of variability and outliers on our analyses by calculating medians and IQRs instead of means and standard deviations, and by using non-parametric tests. The long duration of the experiment may have contributed to the variability (e.g. via invisible damage to the probe), as the number of outliers seems to increase over time. However, we did not notice any probe damage after the experiments. Some samples were lacking due to temporary obstruction of the flow. This is generally thought to be followed by a temporary increase in dialysate concentrations. Of note, outliers showed no obvious correlation for adenosine and AMP within samples. Although we took all reasonable precautions to prevent this, part of the variation might be explained by pollution of the samples. On some of the chromatograms small shoulders were observed on the adenosine peak. Storage at –80°C and transport from Amsterdam to Helsinki would not have improved the sample quality.

Between-subject variation may be explained by individual differences. However, genetic variants in adenosine receptor genes as observed in humans [4] probably cannot explain the observed variation in outbred Wistar rats. The observed variation within subjects can also reflect actual fluctuations in adenosine and AMP concentrations within the mPFC over time.

While adenosine is a neurotransmitter [1], it can also be released by glia, which could add to the variability in concentrations. The glial contribution to extracellular adenosine may also derive from breakdown of ATP [45]. Decay of adenosine in the brain extracellular space is also regulated by glial cells [46]. Glial release is especially important when measuring with microdialysis, as implantation of the probes induces gliosis up to 200–300 μm from the probe [47]. Halassa et al. hypothesized that the adenosine increases associated with prolonged wakefulness result from gliotransmission [48]. They inhibited gliotransmission, which decreased accruing sleep pressure and counteracted the cognitive deficits related to sleep loss in transgenic mice.

We performed a systematic review and meta-analyses on comparable data from the literature to quantitatively summarise all the available evidence. The presented meta-analysis is the first meta-analysis of a neurochemical outcome after sleep deprivation in experimental animals. It includes eight papers retrieved from a preceding SR [28]. It shows that, in the basal forebrain, extracellular adenosine increases during sleep deprivation. For the cortex, the available results are so far inconclusive; by now four different cortical regions (lateral gyrus [26], frontal associative cortex [27], cingulate cortex [27], and mPFC [this study]) have only been measured once. To the best of our knowledge, our study may be the first to report AMP levels measured in animals with microdialysis during and after sleep deprivation; our systematic review did not retrieve any other studies. Of note, we excluded studies on cyclic AMP (e.g. [49,50]), but none of the studies on cyclic AMP retrieved by our search were on sleep deprivation.

The heterogeneity between the included studies in our meta-analysis is high. For the basal forebrain, this can partially be explained by differences in the duration of the sleep deprivation. The two studies implementing the longest sleep deprivation [17,27] show the largest increases in adenosine. The sleep fragmentation studies all fall within the range of the gentle handling studies, indicating that the type of sleep deprivation may not be that relevant for the BF adenosine accumulation. The same can be stated for species, based on the study in cats. Differences in the experimental design, microdialysis methods and other experimental set-up probably explain the remaining heterogeneity. We did not analyse these experimental factors in detail. The BF is a constellation of subcortical structures stretching out in the rostrocaudal direction on the ventral side of the brain. It comprises the nucleus accumbens, nucleus basalis magnocellularis, diagonal band of Broca, substantia innominata, medial septal nuclei and ventral pallidum. Because its nuclei are chemically heterogeneous [8], small differences in probe location can have large effects.

For the cortex, the most sensible explanation of the heterogeneity between the two studies is a difference in probe location and/or species; one of the studies analysed extracellular adenosine in area 17 of the lateral gyrus in cats [26]; the other in the frontal associative cortex in Wistar rats [27].

We did not analyse publication bias, as the number of included studies was considered too low for reliable results. It is likely that small explorative studies with limited power have not been published. Publication of all studies, including the underpowered ones, should be encouraged to make the data available for future meta-analyses.

Our small meta-analysis shows a convincing increase of extracellular adenosine concentrations in the basal forebrain during sleep deprivation. It is not conclusive on effects in the cortex. Further primary studies are still warranted. Future systematic reviews and meta-analyses of extracellular adenosine in different brain regions and species during natural sleep could provide additional information on the neuroanatomical organisation of the sleep-wake cycle.

## Data Accessibility Statement

Data in this publication have not yet been made publicly available. Data included in the systematic review are already in the public domain. Provided data from the microdialysis experiment are sufficient for inclusion in future meta-analyses. Full data will be made available for reuse to individual scientists upon reasonable requests.
